# Acupuncture for Pediatric Pain

**DOI:** 10.3390/children1020134

**Published:** 2014-08-21

**Authors:** Brenda Golianu, Ann Ming Yeh, Meredith Brooks

**Affiliations:** 1 Department of Anesthesiology and Pain Medicine, Pediatric Anesthesiology, Stanford University, 300 Pasteur Dr. Stanford, CA 94304, USA; 2 Pediatric Gastroenterology, Stanford University, 750 Welch Road, Suite 116, Palo Alto CA 94304, USA; E-Mail: annming@stanford.edu; 3 Department of Anesthesiology and Pain Medicine, Pediatric Anesthesiology, Stanford University, 300 Pasteur Dr. Stanford, CA 94304, USA; E-Mail: mrbrooks@stanford.edu

**Keywords:** acupuncture, pediatric, pain, integrative medicine, review, acupressure, laser acupuncture

## Abstract

Chronic pain is a growing problem in children, with prevalence as high as 30.8%. Acupuncture has been found to be useful in many chronic pain conditions, and may be of clinical value in a multidisciplinary treatment program. The basic principles of acupuncture are reviewed, as well as studies exploring basic mechanisms of acupuncture and clinical efficacy. Conditions commonly treated in the pediatric pain clinic, including headache, abdominal pain, fibromyalgia, juvenile arthritis, complex regional pain syndrome, cancer pain, as well as perioperative pain studies are reviewed and discussed. Areas in need of further research are identified, and procedural aspects of acupuncture practice and safety studies are reviewed. Acupuncture can be an effective adjuvant in the care of pediatric patients with painful conditions, both in a chronic and an acute setting. Further studies, including randomized controlled trials, as well as trials of comparative effectiveness are needed.

## 1. Introduction

Chronic pain is a growing problem in children with prevalence as high as 30.8% in some epidemiological studies [1]. The predominant types of pain include headache (60.8%), abdominal pain (43.3%), limb (33.6%), and back (30.2%), and pain symptoms have been found to be associated with many lifestyle restrictions including sleep problems, inability to pursue hobbies, school absences, and inability to meet with friends. Pediatric pain clinics offer many integrative and complementary services including biofeedback (33%), acupuncture (24%), massage (20%), gratitude journals (16%), yoga and tai chi (8%) [2]. In other studies of pediatric headache conducted in Italy and Germany, Complementary Alternative Medicine (CAM) therapies were utilized by 76% and 75.7% of patients respectively [3,4]. Thus, a thorough understanding of the basis and efficacy of these therapies is warranted to help our patients better navigate the extensive selection of therapies, as well as integrate them with conventional medical treatment strategies.

## 2. Physiology of Chronic Pain

The physiology of chronic pain has evolved from a direct stimulus response model to one of an integrated complex neural network response. Following tissue injury, there is an immediate release of local inflammatory mediators, including prostaglandins, histamine, bradykinin and other factors, which lead to a localized tissue response, consisting of localized swelling, pain, and edema. Furthermore, they sensitize the primary neurons such that a subsequent stimulus is perceived as even more painful. The development of chronic pain involves long term potentiation of peripheral nerves, molecular changes in the spinal cord and central nervous system, [5] as well as alteration in how various areas of the mid brain and limbic system, including structures such as insula, prefrontal cortex, anterior cingulate, thalamus, often termed the “pain matrix”, connect to one another. These changes can be observed using functional MRI studies. Improvement in pain is often correlated with the return of normalized connectivity in these areas [6,7].

## 3. Acupuncture: Introduction, Mechanisms of Action, and Clinical Research

### 3.1. Introduction

Acupuncture has been practiced in China for at least 3000 years. The basic principles of acupuncture revolve around the concept of “Qi”, or energy of the body. When this energy is not flowing correctly, it is believed to lead to illness and disease. In the traditional setting acupuncture is frequently utilized as a preventative therapy or an intervention early in the course of illness.

In the pediatric population,, standard medical therapies for chronic pain conditions often carry a significant side effect profile. Side effects of frequently used pharmacological agents for chronic pain include sedation, dizziness, tolerance, nausea, constipation, as well as depression and suicidal ideation. As a result, many parents and children are especially interested in trying acupuncture and other CAM therapies due to their relatively low risk profile.

### 3.2. Acupuncture Mechanisms of Action

A challenging area of research has involved understanding the mechanisms of acupuncture action and how it relates to pain. Although much research has been performed in the last several decades and many basic scientific studies have outlined various physiologic pathways which are activated by acupuncture, a complete synthesis of the mechanism of action of acupuncture is still elusive [8]. Many areas of active research can be identified, and within each hundreds of studies are found, however we will mention three theories that are particularly noteworthy regarding the mechanisms of action of acupuncture: molecular mechanisms, physiologic changes found at acupuncture point locations, and central nervous system changes in brain connectivity. Principal among the molecular studies is the work of JS Han and colleagues, who over several decades explored how acupuncture stimulated the release of endogenous endorphins [9]. One of their most significant findings is that low frequency (2–10 Hz) electrical stimulation of acupuncture points leads to increased endorphin release, while high frequency stimulation (100 Hz) leads to increased dynorphin release. Other studies describe the local changes found at acupuncture points, including connective tissue changes, and changes in electrical resistance at acupoints. For example, Langevin identified changes in connective tissue that occur with the physical act of needle placement [10], while Anh *et al.* explored the changes in skin resistance at distal acupuncture points and found that changes in these responses correlated with clinical response in adolescent girls with pelvic pain [11].

A number of fMRI studies have shown changes in brain blood flow following acupuncture treatment, and normalization of activity in areas of the limbic system, as well as areas of the “pain matrix” [12,13,14]. An excellent review of acupuncture basic mechanisms was performed by Wang *et al.* [15].

### 3.3. Challenges in Acupuncture Clinical Research

In 1997, the NIH conducted a Consensus meeting, where it was determined that sufficient evidence was present to conclude that acupuncture is effective for a number of conditions including nausea and vomiting related to chemotherapy, adult postoperative pain and postoperative dental pain. Additional promising indications included tennis elbow, dysmenorrhea, stroke rehabilitation, and the treatment of addiction [16]. Since that time many additional studies have supported the use of acupuncture in a number of types of painful conditions [17,18].

Some studies, such as the back pain trial in Germany, showed an improvement in both the active and the sham acupuncture arms as compared to standard care, suggesting that some of the acupuncture effect may not depend on the specificity of the points utilized [19]. There is significant controversy in the acupuncture literature about how this matter should be interpreted, as the sham controls in many studies have shown clinical efficacy. One explanation is that since we do not know the exact mechanisms of pain relief of acupuncture, the sham controls, which involve active needle placement in alternate locations, are themselves having a therapeutic benefit. Secondly, the active acupuncture treatment that is performed as part of an RCT may have limitations, as it does not allow individualization of treatment, a necessary ingredient of acupuncture therapy. Thus the finding in many studies that active acupuncture and sham acupuncture appear to both be efficacious remains an interpretive challenge in current acupuncture research, and until this phenomenon is better understood and an appropriate, therapeutically inactive but believable sham treatment can be designed, there has been a call for different types of studies, such as comparative effectiveness trials, which compare the acupuncture therapy against standard therapies, to continue to inform clinical practice [20]. MacPherson *et al*., in a meta-analysis of published studies, determined that positive trials were associated with more needles and more frequent treatments, suggesting that dosing of acupuncture may also be important [21].

## 4. Pediatric Pain

Acupuncture can be a useful adjuvant in the care of pediatric patients with painful conditions, both in the chronic and acute setting. In a study on the feasibility of acupuncture/hypnosis intervention, 33 children were offered acupuncture together with a hypnosis session while needles were in place. Treatments were highly acceptable (only 2 patients refused). Both parents and children reported decrease in pain (4.38 (*p* < 0.0001)), and improvement in function (2.62 (*p* = 0.014)) [22]. Lin *et al.* investigated the efficacy of acupuncture for children and adolescents with chronic pain. Fifty-three patients between 2 and 18 years of age, with a variety of painful conditions underwent pain assessment before and after acupuncture treatment. The average duration of pain improvement was 3 days, suggesting that continued acupuncture was needed for longer term improvement [23].

Conditions commonly treated in pediatric pain, including headache, abdominal pain, fibromyalgia, juvenile arthritis, complex regional pain syndrome, cancer pain, as well as perioperative pain studies will be discussed. Due to the paucity of acupuncture research in the pediatric literature, adult studies will also be reviewed in each section, as they often serve as the clinical basis for the development of treatment regimens. Lastly, areas in need of further research of acupuncture in children will be identified. 

### 4.1. Procedure Consent, Acceptance and Safety of Acupuncture

#### 4.1.1. The Procedure of Acupuncture

Informed consent is obtained from the parents prior to initiation of acupuncture treatment. The type of acupuncture is explained to both the parent and child, along with possible complications, which may include bleeding, infection, or increased pain. More serious complications have occurred (see below) and may be related to needling technique or patient condition. A refusal of the parent or child to receive acupuncture should be a contraindication. Pediatric patients often require more gentle introduction to the needling process, to allow them to grow more accustomed to the idea, trust the practitioner, and observe for themselves that the needles usually do not cause excessive discomfort. Lin *et al*. showed that while 53% of children were initially apprehensive of acupuncture needles, following their first needle 64% felt it did not hurt, and furthermore would recommend it to someone else.. Acupuncture treatment will usually consist of a series of treatments, usually 6–10, to determine if acupuncture intervention is helpful for the patient’s condition. In a study by Kemper of adolescents receiving acupuncture for their pain, 67% reported the therapy as pleasant, and 70% reported that it had helped their pain [24]. Children were moderately receptive to acupuncture therapy for their chronic pain symptoms [25]. Parental experience with acupuncture, regardless of its perceived efficacy, appears to play in a role in parents’ consideration of acupuncture for their child [26]. Parental stress was found to decrease along with child discomfort following acupuncture therapy for the child [27]. 

#### 4.1.2. Acupuncture Safety

In an excellent review of the safety of acupuncture in the pediatric population, Adams *et al.* screened a total of 9537 references, and identified 37 studies in the international literature that satisfied criteria for inclusion. Described complications included infection, and 1 case each of cardiac rupture, pneumothorax, and other effects, likely related to direct needle organ penetration. Additional mild adverse effects included pain, bruising, and worsening of symptoms. The study concluded that acupuncture is safe when performed by appropriately trained practitioners [28]. Yates *et al*. showed that non-invasive electrical stimulation at acupuncture points during a routine heel stick was well tolerated in healthy neonates [29].

#### 4.1.3. Headache

In the adult literature, several studies examine acupuncture as a prophylactic treatment. Allais *et al.* performed a randomized trial of 160 women migraine sufferers who received weekly acupuncture for 2 months, followed by monthly acupuncture for an additional 4 months. Decreased headache severity, as well as decreased medication usage, was statistically significant at all time points analyzed [30]. Diener *et al.* performed a large multicentre randomized study of 443 patients randomized into three groups—verum acupuncture, sham acupuncture (termed New Western acupuncture by the researchers), and standard medical care. Following 10 weekly acupuncture treatments, headache frequency and severity was measured at 26 weeks after baseline. Although the results were statistically significant, the effect size may have been diminished by the long time lapse between last acupuncture treatment and pain assessment [31]. In another study of migraine sufferers, the study intervention was delivered in a standard clinical format, without randomization. Study results showed that acupuncture had a measurable clinical effect, which was greater than that seen in a randomized format [32]. Finally two large Cochrane Collaboration Reviews concluded that although it appeared that specific point selection was not as important as had previously thought, acupuncture should be considered as a treatment option for patients needing prophylactic treatment for migraine or tension headaches [33,34].

In the pediatric population, a randomized trial of 22 children with migraine showed in the true acupuncture group the migraine frequency decreased from 9.3 (±1.6) to 1.4 (±0.6), and intensity decreased from 8.7 (±0.4) to 7.8 (±0.6) following 10 weekly sessions [35]. Gottschling *et al.* used a non-invasive technique, laser acupuncture, in a randomized trial of 43 children with either migraine or tension type headache. The mean number of headaches per month decreased by 6.4 days in the treatment group, and by 1.0 day in the placebo group (*p* < 0.001). Secondary outcomes of headache severity were likewise decreased, and were statistically significant at all time points [36]. Further studies are needed exploring the use of acupuncture as a prophylactic agent in the pediatric population, as well as studies which combine the use of a low dose prophylactic medication together with acupuncture. 

#### 4.1.4. Abdominal Pain

Abdominal pain can be due to a multitude of diagnoses, and a full review of these is beyond the scope of this work. We will first review the available adult literature on the use of acupuncture in the management of pain related to irritable bowel syndrome (IBS), a functional disorder characterized by chronic or recurrent abdominal pain or discomfort which is associated with disturbed bowel function, and feelings of abdominal distention and bloating [37]. Chao *et al.* performed a meta-analysis of 6 randomized placebo controlled studies [38]. Although five of the studies did not show statistically significant improvement, the one positive study in the group was a relatively large study [39], and thus the overall meta-analysis provided support for the use of acupuncture. A Cochrane review of acupuncture for IBS showed no benefit from acupuncture as compared to credible sham controlled therapies, however a comparative effectiveness trial of acupuncture compared to two antispasmodics (pinaverium bromide and trimebutin maleate) showed acupuncture as more effective than these standard therapies for IBS [40]. In a pediatric trial on children with intermittent abdominal pain that compared hand acupuncture to no treatment, hand acupuncture was shown to be effective [41]. As the trial was not blinded, however, the results are difficult to interpret. Further studies are needed to delineate whether there is clinical benefit of acupuncture in this condition, the necessary dose or frequency and duration of acupuncture treatment required, as well as possible mechanisms of action.

#### 4.1.5. Pediatric Fibromyalgia

Pediatric fibromyalgia is a poorly understood condition that affects 1%–6% of populations studied, and includes symptoms of general fatigue, disordered sleep, severe myofascial pain, and abdominal dysregulation [42]. The mechanisms of fibromyalgia pain are not fully understood, but may include genetic, anatomic, metabolic and psychosocial factors [43]. Acupuncture was found to change cortical responses to painful stimuli in fibromyalgia patients, suggesting a complex inhibitory modulation may be active in the central nervous system in fibromyalgia patients [44].

In the adult population, acupuncture studies have demonstrated that acupuncture may be a significant adjunct in the care of fibromyalgia patients, and is superior to standard care alone [45]. It is noted however that sham acupuncture likewise appeared more effective than standard care, making it difficult to determine the specificity of the acupuncture needle placement in this study. Although acupuncture is routinely offered and utilized as a CAM therapy in pediatric pain clinics by patients with fibromyalgia, to date there have been no randomized controlled studies in the pediatric population [46]. Future acupuncture research in children with fibromyalgia should examine its benefits, comparative effectiveness, mechanism of action and necessary dose.

#### 4.1.6. Acupuncture for Juvenile Arthritis

Children with juvenile idiopathic arthritis (JIA) and other rheumatological conditions utilize CAM therapies regularly, with frequencies between 34% and 92% [47]. Factors associated with CAM use include longer disease duration, presence of more than one illness, previous CAM use by parents themselves, and parents’ perception that medications are not helping [48,49]. In adults, Berman *et al.*, in a landmark randomized controlled study of osteoarthritis, found that the true acupuncture group experienced a greater improvement in WOMAC function at 8 weeks than the sham acupuncture group [50]; however, a subsequent meta-analysis of nine studies showed short-term benefits compared to sham, and clinically relevant benefits relative to wait-list control [51]. Zanette *et al.* studied 40 adults on a stable medication regimen with uncontrolled rheumatoid arthritis in a randomized controlled study. There was significant improvement in patient and physician global assessment and in physician assessment of disease activity in the acupuncture group, however the primary outcome of 20% improvement in ACR criteria was not statistically significant [52]. There are no studies in the literature evaluating the effect of acupuncture on juvenile arthritis. Studies are needed to explore the efficacy of acupuncture in pediatric rheumatological conditions.

#### 4.1.7. Pelvic Pain

Acupuncture has been shown to be effective in pain related to dysmenorrhea in multiple adult randomized clinical trials. Kiran *et al*. showed that acupuncture was as effective as NSAIDS in a small, randomized trial [53]. A Cochrane review concluded that acupuncture may be effective for dymenorrhea, however more studies were needed [54]. A study of acupressure in adolescents with dysmenorrhea showed a decrease in pain in the experimental group that was statistically significant (*p* < 0.002) compared to the sham acupressure group [55]. Sham acupressure also had some effect of improved analgesia, however, thus a third control of standard care or other relatively inert control would have been beneficial and may have shown an even greater clinical significance. In a small randomized sham controlled pediatric study on pelvic pain due to endometriosis, Wayne *et al.* showed that participants experienced a 4.8 (SD 2.4) point reduction on a 11 point scale after 4 weeks, which differed significantly from the control group who experienced an average reduction of 1.4 (SD2.1) (*p* = 0.004). Reduction in pain persisted 6 months after intervention; however, after 4 weeks the differences were not clinically significant, suggesting continued acupuncture may be needed for a more prolonged therapeutic effect [56]. Further research is needed in pediatric patients with pelvic pain focusing on comparative effectiveness with standard treatments, dose efficacy and mechanism of action.

#### 4.1.8. Complex Regional Pain Syndrome

Little information exists regarding the treatment of Complex Regional Pain Syndrome (CRPS) with acupuncture. Several case reports in adult military personnel showed benefits in shoulder and hand CRPS following scalp acupuncture [57]. An adult case study involving laser acupuncture and small subcutaneous needles also proved to be effective [58]. In children, one case study describes the use of electrical stimulation at acupuncture points, while another describes the use of acupuncture in three pediatric patients leading to clinical improvement [59,60]. Further studies in children with complex regional pain syndrome, including scalp acupuncture, electrical stimulation and other specific protocols are needed to delineate whether there is measurable and reproducible benefit.

#### 4.1.9. Acupuncture for Cancer Pain

The management of cancer pain presents a complex challenge for the oncologist and the pain practitioner. Pain may be due to acute tissue invasion, inflammation, bone pain, neuropathic pain, or a combination of causes [61]. In addition, cancer patients suffer from other chemotherapeutic side effects including nausea and vomiting. CAM therapies are frequently utilized by this population. A prevalence study of CAM use in children and adolescents with cancer in Germany found that 29% of children and 36% of adolescents used CAM therapies. Reasons for using CAM included strengthening the immune system, reduction of therapy-related side effects, and desire to increase healing chances [62]. Acupuncture was used by 12% of children and 17% of adolescents. In a randomized trial of acupuncture to alleviate chemotherapy related nausea and vomiting due to highly emetogenic chemotherapy, the need for rescue anti-emetics was significantly lower in the acupuncture group than in the control group (*p* < 0.001) [63]. Episodes of vomiting were likewise lower in the treatment group (*p* = 0.01). Acupuncture was found to be safe in patients with thrombocytopenia due to chemotherapy [64]. 

#### 4.1.10. Acupuncture for Acute Pain

Two studies have explored the use of acupuncture in the treatment of acute post-operative pain in pediatric patients. Wu *et al.* reported a case series of 20 pediatric patients, aged 7 months to 18 years, who had undergone various surgeries [65]. Acupuncture was performed in the post-operative period. Pain scores declined immediately following acupuncture, as well as at 24 hours after acupuncture was performed. The acupuncture was well tolerated, and 85% of parents reported they would pay for acupuncture “out of pocket” if not covered by insurance. This study was not controlled, however. 

Lin *et al.* performed a randomized trial examining pain and delirium in pediatric patients following tympanostomy surgery [66]. Sixty children were randomized to acupuncture or sham acupuncture following induction of anesthesia. Statistical differences in pain scores were observed in post anesthesia care unit at each five minute time point. Median pain scores were between 2 and 4 points lower than the sham acupuncture group. Postoperative agitation was also decreased, and showed statistical significance at all time points. Additional studies are needed to explore the magnitude of the clinical effect of various acupuncture treatments, duration of action, as well as post-operative opioid use and surgical recovery time.

#### 4.1.11. Acupuncture for Delirium, Agitation, Withdrawal and Emotional Conditions

Several studies exist on the use of acupuncture on post-traumatic stress disorder. Hollifield *et al.* found that acupuncture was as effective as cognitive behavioral therapy, and Zhang *et al.* found that acupoint stimulation together with cognitive behavioral therapy was more effective than cognitive behavioral therapy alone [67,68]. To date, no studies have been identified examining the efficacy of acupuncture in children with post traumatic stress disorder. Several studies specifically address the effect of acupuncture interventions on withdrawal and agitation in children. Wang *et al.* found that acupressure reduced anxiety in children undergoing anesthesia [69]. Kundu *et al.* performed a retrospective review of pediatric patients who had postoperative agitation on a previous anesthetic, and received acupuncture with a subsequent anesthetic [70]. 10 patients (83%) did not exhibit symptoms of agitation, while 2 (17%) exhibited milder symptoms but were able to communicate their distress. In a study of neonates with Neonatal Abstinence Syndrome (NAS), the addition of auricular acupressure beads did not result in a different clinical course, though there was a suggestive trend toward less pharmacological support in the acupressure group [71].

#### 4.1.12. Non-Invasive Transcutaneous Electrical Acupoint Stimulation (TEAS)

As some pediatric patients, as well as some adults, may have a fear of needles, the use of transcutaneous electric acupoint stimulation (TEAS) may provide an alternative to conventional acupuncture treatments. The effectiveness of TENS may be mediated by alpha 2A adrenergic receptors [72]. A number of studies suggest that such stimulation may be clinically effective. Wang *et al.* showed that TEAS reduced intraoperative remifentanil consumption and alleviated post-operative side effects following sinusotomy [73]. TEAS was also applied by Kabalak for the management of postoperative vomiting following tonsillectomy in children in a randomized study [74]. In another randomized double blinded perioperative study, TEAS was applied at the start of surgery in patients undergoing pediatric cardiac surgery [75]. Duration of ventilation, and length of stay in ICU were significantly lower in the active treatment group. In addition, cardiac troponin, a measure of damaged cardiac tissue, was significantly lower in the active treatment group. As TEAS is a non-invasive technique, it has significant promise as a potential treatment in children.

#### 4.1.13. Non-Invasive Laser Acupuncture

Laser acupuncture may also be utilized in the clinic for management of pain and discomfort in the pediatric patient. In addition to the headache study as mentioned above by Gottschling *et al.*, others have demonstrated clinical utility. Ferreira *et al.* described a case report of improved trismus as a sequelae of medulobastoma in a child [76]. Other non-invasive therapies offered in pediatric pain clinics include moxibustion, cupping and acupressure magnets [77].

### 4.2. Cost-Effectiveness of Acupuncture Therapy

This is a relatively new area of research, however, an National Health Service (NHS) analysis of low back pain studied in 239 patients, with 159 randomized to acupuncture and 80 to usual care, found that the addition of acupuncture to standard care led to improved pain both immediately after the treatments as well as at 24 months. Though the cost of delivering acupuncture in addition to standard care was an increase of expenditure, this cost was more than balanced by a reduction in lost days of work, and decreased medical spending in other areas including hospitalization, general practitioner or other outpatient visits. This suggests an overall societal benefit to supporting the provision of acupuncture services [77]. Further research is needed examining the cost-effectiveness of acupuncture in children in relation to lost parental work days, cost of medications, and other therapeutic interventions.

## 5. Conclusions

Acupuncture techniques, both invasive and noninvasive, can be an important adjuvant in the care of the pediatric patient with chronic pain. Acupuncture should be performed by a trained professional, and should be incorporated in a multidisciplinary program of treatment, after appropriate workup has been performed. The evidence suggests that it is a safe and cost-effective treatment modality for pediatric pain. Further research is needed regarding the specific and non-specific effects of acupuncture, as well as mechanisms of action, dosing and frequency related to various painful conditions in pediatric patients.
